# Secure UPnP Resource Discovery Using a PUF-Assisted Hardware Accelerator for IoT

**DOI:** 10.3390/s26175410

**Published:** 2026-08-27

**Authors:** Kasem Khalil

**Affiliations:** 1 Electrical and Computer Engineering Department, University of Mississippi, Oxford, MS 38677, USA; kmkhalil@olemiss.edu; 2 Electrical Engineering Department, Assiut University, Assiut 71515, Egypt

**Keywords:** IoT, electronics, resource discovery, UPNP, security, PUF, hardware accelerator

## Abstract

Universal Plug and Play (UPnP) is widely used for resource discovery in internet of things and smart-edge environments because of its lightweight and decentralized operation. However, conventional UPnP and Simple Service Discovery Protocol (SSDP) mechanisms expose static identifiers and service metadata, making them vulnerable to spoofing, replay, unauthorized resource enumeration, device fingerprinting, and long-term traffic correlation. Existing software-based authentication methods rely on stored credentials and do not protect discovery privacy, while conventional Arbiter Physical Unclonable Functions (PUFs) remain vulnerable to machine-learning modeling attacks and are typically used only for device authentication. This paper presents a novel Recursive Hybrid Entropy PUF (RHE-PUF) and a privacy-preserving secure UPnP discovery architecture. The proposed RHE-PUF introduces recursive adaptive delay propagation, entropy injection, feed-forward coupling, and multi-path timing diversification to increase challenge–response nonlinearity and modeling resistance. Its responses are used to generate dynamic ephemeral identities, authenticate devices anonymously, and encrypt SSDP service advertisements without exposing permanent device identifiers. The complete framework was implemented on a Xilinx Spartan-7 FPGA and evaluated under realistic UPnP discovery and attack scenarios. The RHE-PUF achieved 49.31% uniqueness, 98.14% reliability, 50.22% uniformity, and 98.91% entropy. The implementation operated at up to 192 MHz with 0.84 W dynamic power, 0.88 µs authentication latency, and 13.4 ms secure discovery delay. The strongest deep-neural-network modeling attack achieved only 58.27% prediction accuracy. Replay and spoofing attack success rates were reduced to at or below 1.1% and 0.8%, respectively, while long-term tracking probability remained below 13%. These results demonstrate that the proposed joint hardware-security and privacy-preserving discovery framework provides resource-efficient authentication, anonymous UPnP resource discovery, and resistance to network and machine-learning attacks.

## 1. Introduction

The rapid growth of Internet of Things (IoT) systems has increased the use of lightweight service-discovery protocols in smart homes, industrial automation, healthcare, transportation, and edge-computing environments [1,2,3,4,5]. A massive number of interconnected devices continuously exchange data and services through lightweight discovery and communication protocols [6,7,8]. Among these protocols, Universal Plug and Play (UPnP) is widely adopted because it allows heterogeneous devices to advertise services and discover neighboring resources without centralized configuration. UPnP discovery primarily relies on the Simple Service Discovery Protocol (SSDP), which distributes multicast advertisements containing device and service information. The lack of native authentication and privacy protection in conventional SSDP exposes UPnP systems to spoofing, replay, unauthorized service enumeration, metadata leakage, and device tracking. In particular, static device identifiers and unprotected service descriptors allow an observer to associate repeated advertisements with the same physical device, infer its capabilities, and correlate its activity over extended periods. Therefore, securing UPnP discovery requires more than verifying whether a device is legitimate; it also requires preventing identity exposure, metadata disclosure, and traffic correlation during the discovery process [9,10,11,12].

Conventional software-based authentication mechanisms remain insufficient for protecting resource-constrained IoT devices due to several limitations, including secret key storage vulnerabilities, software cloning attacks, firmware extraction risks, and high cryptographic overhead [13,14,15,16]. Recent large-scale cyberattacks exploiting insecure UPnP-enabled IoT devices have demonstrated the critical need for lightweight hardware-rooted security architectures capable of providing intrinsic trust establishment and privacy preservation directly at the device level.

Physical Unclonable Functions (PUFs) have recently emerged as promising hardware security primitives for low-overhead authentication and device fingerprinting [16,17,18,19,20,21,22]. PUFs exploit uncontrollable manufacturing process variations to generate unique and unclonable challenge–response pairs (CRPs) for each hardware instance. Unlike conventional cryptographic key storage mechanisms, PUF-based systems eliminate the need for permanent secret storage and provide intrinsic hardware-rooted identity generation. Among various PUF architectures, Arbiter PUFs are particularly attractive for FPGA-based low-overhead authentication due to their compact implementation and low hardware complexity. However, conventional Arbiter PUF architectures still suffer from several critical limitations that restrict their applicability in practical IoT security environments. Most existing Arbiter PUFs exhibit partially linear delay propagation behavior, making them vulnerable to machine learning modeling attacks including Logistic Regression (LR), Support Vector Machine (SVM), Evolutionary Strategies (ES), and Deep Neural Networks (DNNs) [23,24,25,26,27]. Once an attacker collects sufficient CRPs, the internal propagation characteristics of the PUF can be approximated with high prediction accuracy. Moreover, existing PUF-based authentication frameworks primarily focus on device authentication while neglecting privacy-aware resource discovery and anonymous communication requirements in UPnP ecosystems.

In addition, existing UPnP security frameworks mainly rely on centralized trust architectures, software-managed certificates, or computationally intensive cryptographic operations that are unsuitable for resource-constrained IoT edge environments [2,10]. Most prior works fail to jointly address hardware authentication, anonymous service discovery, metadata protection, anti-tracking capability, and resistance against machine learning attacks within a unified framework. Consequently, a significant research gap still exists in designing lightweight FPGA-oriented architectures capable of simultaneously achieving secure UPnP discovery, privacy preservation, and strong anti-modeling security.

**Figure 1:**
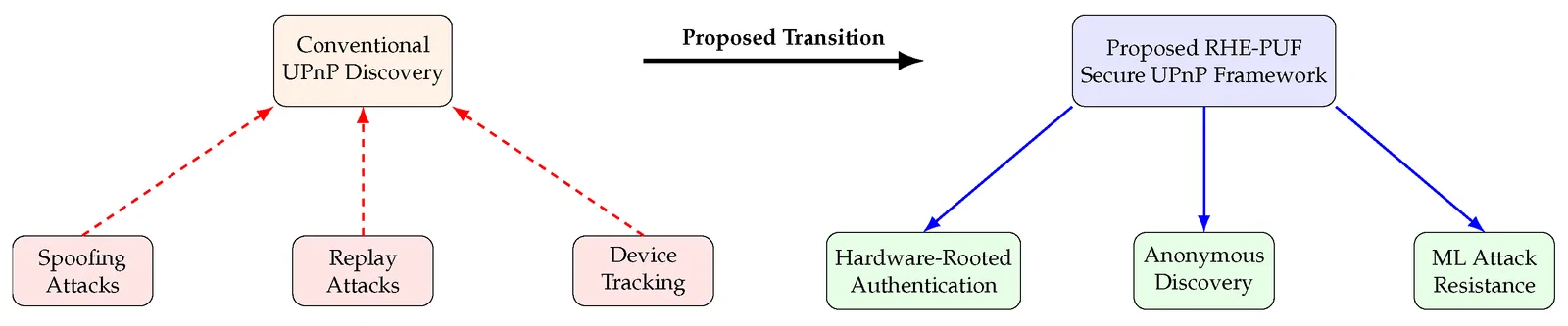
Transition from conventional UPnP discovery vulnerabilities to the proposed RHE-PUF-based protection mechanisms.

To address these challenges, this paper proposes a unified FPGA-based privacy-preserving secure UPnP framework driven by a Recursive Hybrid Entropy Physical Unclonable Function (RHE-PUF). The proposed architecture introduces a fundamentally new recursive entropy-enhanced Arbiter PUF structure that combines adaptive delay propagation, recursive feed-forward coupling, entropy injection nodes, and multi-path timing diversification to substantially increase CRP nonlinearity and unpredictability. Unlike conventional Arbiter PUFs, the proposed RHE-PUF continuously perturbs internal propagation behavior through recursive adaptive timing paths, thereby significantly improving resistance against machine learning approximation attacks. Furthermore, the proposed framework integrates the RHE-PUF into a complete privacy-aware UPnP discovery architecture capable of generating dynamic ephemeral identities and anonymous discovery tokens for secure SSDP communication. The proposed framework eliminates static device identifiers during discovery operations and introduces encrypted anonymous service advertisements to prevent device fingerprinting, replay attacks, traffic correlation, and metadata leakage. Figure 1 illustrates the fundamental security and privacy limitations of conventional UPnP architectures compared to the proposed hardware-rooted privacy-enhanced framework. The major contributions of this paper can be summarized as follows:

- A novel RHE-PUF architecture is developed using triple-path propagation, adaptive cross-coupling, recursive feed-forward timing, and entropy injection to improve challenge–response nonlinearity and resistance to machine-learning modeling attacks.
- The RHE-PUF is integrated with a privacy-preserving UPnP/SSDP discovery architecture.
- A dynamic ephemeral identity mechanism is introduced to support authenticated discovery without transmitting permanent device identifiers.
- Encrypted SSDP advertisements and protected service enumeration are used to reduce metadata leakage, unauthorized discovery, spoofing, replay, and traffic-correlation attacks.

The remainder of this paper is organized as follows. Section 3 presents the proposed RHE-PUF architecture and privacy-preserving UPnP approach. Section 4 presents experimental results and security analysis. Finally, Section 5 concludes the paper.

## 2. Related Works

Existing studies related to secure IoT resource discovery can be broadly categorized into three groups: software-based authentication, PUF-based authentication, and privacy-aware service discovery. Although each category addresses an important aspect of IoT security, none of them jointly provides PUF-based authentication, resistance to machine-learning modeling attacks, anonymous UPnP/SSDP discovery, and metadata protection.

### 2.1. Software-Based Authentication

Software-based authentication approaches commonly rely on cryptographic keys, certificates, hash functions, and challenge–response protocols to verify the identities of IoT devices [28]. Pereira et al. [29] proposed an authentication and access-control framework for Constrained Application Protocol (CoAP)-based IoT systems to provide secure resource access in constrained environments. Similarly, Aman et al. [30] introduced a lightweight mutual-authentication protocol intended to protect IoT communication sessions against impersonation and replay attacks. These approaches provide important security functions, including mutual authentication, access control, anonymity, and replay protection. However, they primarily depend on software-managed cryptographic credentials, stored secret keys, or centralized trust assumptions. Consequently, they may remain vulnerable to key extraction, firmware compromise, memory disclosure, and device-cloning attacks. More importantly, successful device authentication does not necessarily provide privacy during UPnP/SSDP resource discovery. Even an authenticated device may continue to broadcast static identifiers, manufacturer information, firmware details, and service descriptors, thereby enabling device fingerprinting, unauthorized resource enumeration, metadata leakage, and long-term traffic correlation.

### 2.2. PUF-Based Authentication

PUFs provide an attractive hardware-rooted authentication mechanism by deriving device-specific challenge–response behavior from uncontrollable manufacturing variations in semiconductor devices. He et al. [31] proposed a highly reliable Arbiter-PUF architecture with improved uniqueness and experimentally validated the design on FPGA hardware. Their work demonstrates the effectiveness of FPGA-based PUFs for generating device-specific hardware identities while addressing reliability and uniqueness limitations of conventional Arbiter-PUF structures. Similarly, Ge et al. [32] introduced a challenge-preprocessing structure for Arbiter-PUFs specifically aimed at improving resistance against machine-learning modeling attacks. Their results demonstrate that suitable challenge preprocessing can substantially reduce the ability of learning algorithms to approximate the underlying PUF behavior. Other studies have investigated lightweight hybrid and self-correcting PUF architectures for resource-constrained IoT environments. Anandakumar et al. [33] developed an FPGA-based hybrid PUF architecture that combines different PUF mechanisms to improve the security and performance characteristics of conventional PUF implementations. Agarwal and Joshi [34] proposed a self-correcting PUF for IoT device authentication and experimentally demonstrated improved response reliability using error-correction techniques. In addition, Babaei et al. [35] introduced the Reconfigurable Security Architecture (RESA), which employs PUF-based security mechanisms for FPGA-based IoT devices and investigates resistance against machine-learning attacks. Yoon et al. [36] further investigated heterogeneous PUF-based IoT authentication by combining PUF information with physical-layer characteristics to enhance authentication security. Although these approaches demonstrate the effectiveness of PUFs for hardware-rooted authentication, their primary objectives remain device authentication, PUF reliability, uniqueness, reconfigurability, or resistance against modeling attacks. They do not jointly address privacy-aware UPnP resource discovery, anonymous SSDP advertisements, service-metadata confidentiality, protected resource enumeration, and long-term traffic-correlation protection. Furthermore, the existence of machine-learning-resistant PUF constructions does not by itself guarantee privacy preservation at the network-discovery layer. An attacker may still correlate repeated service advertisements or exploit exposed device and service metadata even when the underlying hardware authentication primitive is secure. These observations motivate the integration of a nonlinear, entropy-enhanced PUF with an anonymous and privacy-aware UPnP discovery architecture, as pursued in the proposed RHE-PUF framework.

### 2.3. Privacy-Preserving Service Discovery

Privacy-preserving service discovery aims to reduce the exposure of device identities, service metadata, network characteristics, and resource relationships during dynamic resource discovery. In conventional IoT discovery environments, service advertisements can expose device types, service descriptions, identifiers, network addresses, and temporal traffic patterns. Even when authentication is employed, repeated discovery messages may allow a passive adversary to associate multiple observations with the same physical device. Consequently, authentication alone does not necessarily prevent device fingerprinting, service enumeration, or long-term traffic-correlation attacks. The PUF-based approaches described in [31,32,33,34] primarily protect the hardware authentication primitive and do not modify the resource-discovery process. Likewise, the reconfigurable FPGA security architecture in [35] and the heterogeneous authentication mechanism in [36] focus primarily on establishing or strengthening device trust rather than providing anonymous UPnP/SSDP resource discovery. Therefore, these approaches do not explicitly provide dynamic anonymous identities for discovery sessions, encrypted service advertisements, privacy-aware resource matching, or protection against long-term correlation of discovery traffic.

The proposed framework addresses this gap by extending the hardware-rooted trust established by the RHE-PUF into the UPnP discovery layer. Following successful PUF validation, the reconstructed hardware-derived secret is combined with a fresh nonce and temporal information to generate a session-dependent ephemeral identity. The identity is subsequently used by the privacy-aware discovery engine rather than exposing a persistent device identifier. In addition, service information is protected before being introduced into the SSDP discovery process, while access-policy verification controls which resources can be disclosed to an authenticated requester. This combination allows the proposed framework to address both hardware-level trust and network-level privacy, which are treated separately in most existing PUF-based IoT authentication architectures.

### 2.4. Research Gap

The three categories of existing work address complementary but incomplete aspects of secure IoT resource discovery. Software-based authentication schemes provide access control and replay protection but rely on stored credentials and do not prevent identity or metadata exposure during SSDP communication. PUF-based authentication schemes provide hardware-rooted identities without permanent secret-key storage, but they mainly focus on authentication and may remain vulnerable to machine-learning modeling attacks. Privacy-preserving discovery schemes protect service information and reduce device tracking, but they generally lack hardware-rooted trust and depend on software-managed cryptographic credentials. Therefore, a research gap remains in developing a unified resource-efficient architecture that simultaneously provides hardware-rooted device authentication without permanent secret-key storage, resistance to machine-learning modeling attacks, dynamic and anonymous device identities, encrypted SSDP advertisements and protected service metadata, protection against replay, spoofing, and unauthorized resource enumeration, and privacy-aware UPnP resource discovery suitable for FPGA-based IoT devices. To address this gap, the proposed framework integrates a RHE-PUF with ephemeral identity generation, encrypted SSDP advertisements, privacy-aware service matching, and adaptive trust verification.

## 3. The Proposed Method

This section presents the proposed hardware-assisted privacy-preserving UPnP security framework based on a novel RHE-PUF. The proposed framework introduces a hardware-rooted trust establishment mechanism that combines adaptive entropy-driven PUF authentication, anonymous discovery tokenization, secure resource advertisement, and privacy-aware service orchestration into a unified UPnP security architecture. Unlike conventional UPnP security solutions that rely on static identifiers, software-managed credentials, or centralized authentication infrastructures, the proposed design establishes a fully decentralized hardware-bound identity generation mechanism capable of producing unclonable, dynamic, and non-transferable device identities.

**Figure 2:**
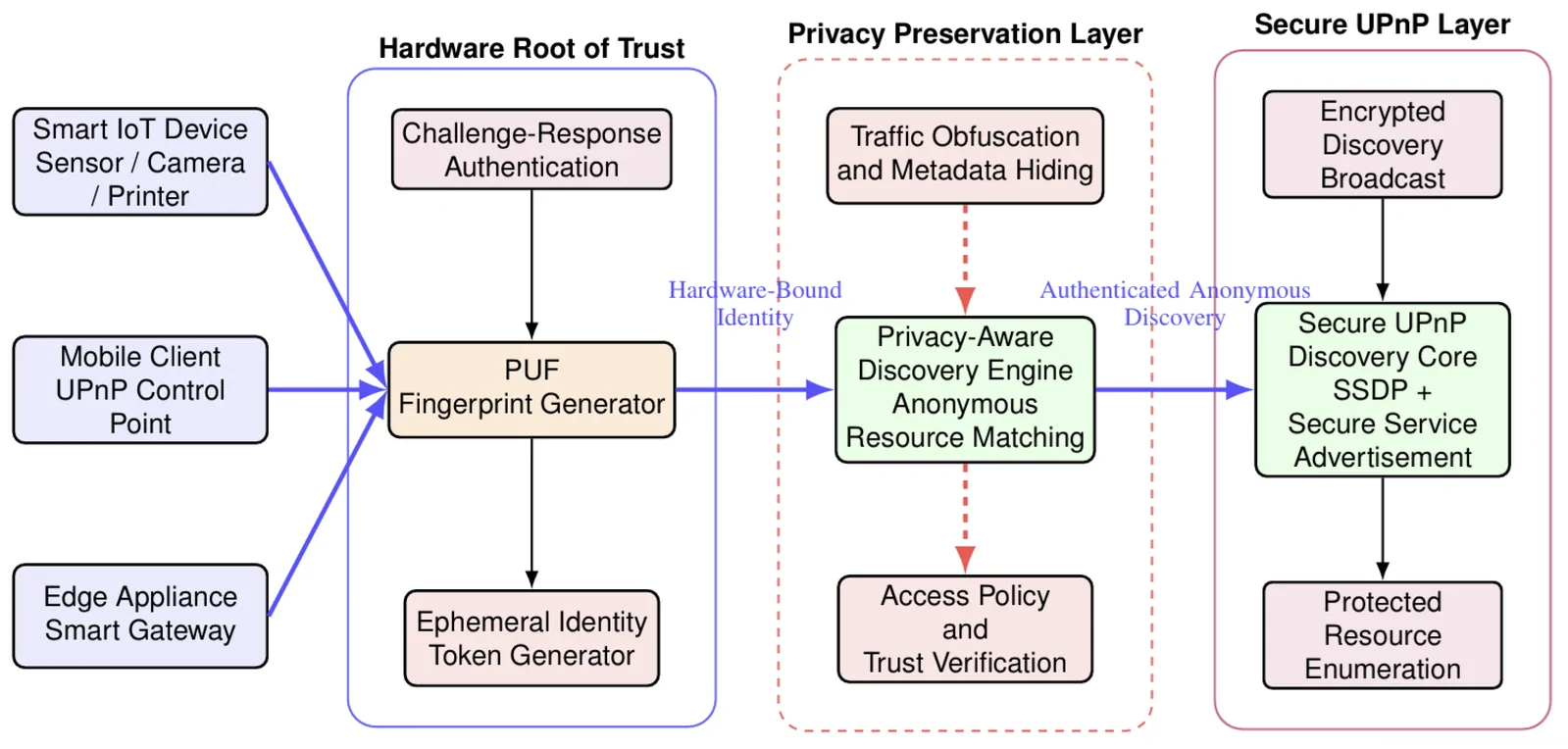
The architecture of the proposed secure UPnP framework.

As illustrated in Figure 2, the proposed framework consists of three tightly integrated layers: the Hardware Root of Trust Layer, the Privacy Preservation Layer, and the Secure UPnP Discovery Layer. The Hardware Root of Trust Layer utilizes the proposed RHE-PUF architecture to generate physically unique CRPs for each participating node. The generated responses are transformed into ephemeral anonymous identities that are processed by the privacy-aware discovery engine before being utilized by the secure UPnP discovery core. This layered architecture enables authenticated yet anonymous service discovery while simultaneously preventing device cloning, metadata leakage, traffic correlation, and unauthorized resource enumeration.

**Figure 3:**
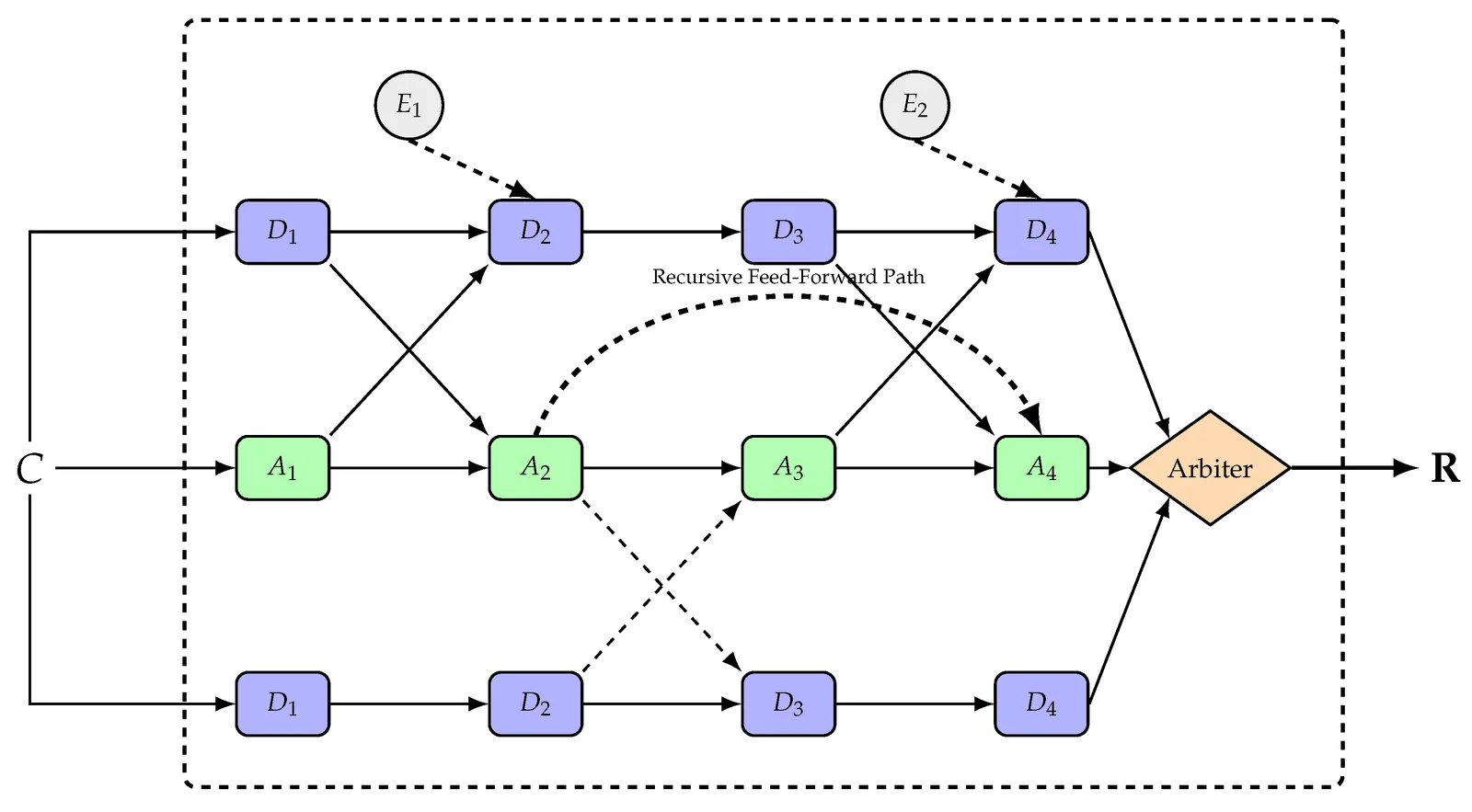
Architecture of the proposed RHE-PUF.

The proposed system begins by assigning each IoT device, mobile client, or edge gateway a hardware-rooted identity generated directly from intrinsic semiconductor manufacturing variations. Unlike software-generated identifiers that can be copied or forged, the proposed hardware identity emerges from uncontrollable nanoscale delay variations inside the RHE-PUF structure shown in Figure 3. Let the challenge vector applied to the PUF be represented as

(1) $$C\in {\left\{0,1\right\}}^{n}$$

where *n* denotes the challenge length. The challenge propagates simultaneously through three cooperative delay paths composed of upper deterministic stages, lower deterministic stages, and a novel adaptive recursive middle propagation path. Due to unavoidable process-induced variations, each propagation path exhibits unique delay characteristics. Consequently, the generated response can be represented as

(2) $$R=f_{\mathrm{RHE}\text{-}\mathrm{PUF}}\left(C,\Delta_{d},\Delta_{r},\Delta_{e}\right)$$

where $\Delta_{d}$ denotes intrinsic propagation delay variations, $\Delta_{r}$ represents recursive adaptive coupling variations, and $\Delta_{e}$ corresponds to entropy-induced perturbation effects. Since these parameters originate from uncontrollable fabrication randomness, reproducing identical responses externally becomes physically infeasible.

The proposed RHE-PUF shown in Figure 3 significantly differs from conventional arbiter PUF architectures by introducing three major innovations. First, the design incorporates a triple-propagation delay structure instead of the traditional dual-path topology. Second, adaptive cross-coupling links dynamically alter signal propagation behavior across neighboring stages. Third, recursive feed-forward entropy reinforcement continuously modifies the internal timing dynamics, thereby increasing CRP unpredictability and resistance against machine learning attacks. The cumulative propagation delay of the proposed architecture can be expressed as

(3) $$\tau_{i}=\sum_{k=1}^{m}d_{k}+\sum_{j=1}^{p}\alpha_{j}+\sum_{q=1}^{r}\epsilon_{q}$$

where $d_{k}$ denotes the challenge-controlled deterministic delay contribution of stage *k*, $\alpha_{j}$ denotes the additional history-dependent delay introduced by adaptive cross-coupling and recursive feed-forward interconnections, and $\epsilon_{q}$ represents a bounded entropy-induced timing perturbation applied by the entropy nodes. The recursive delay term $\alpha_{j}$ captures the influence of earlier propagation states on subsequent stages, while $\epsilon_{q}$ introduces an additional internal timing variation intended to reduce the linear separability of the challenge–response relationship. The entropy contribution is bounded so that it perturbs the relative path delays without dominating the intrinsic process-dependent delay difference, thereby improving nonlinearity while preserving response repeatability. The adaptive recursive delay evolution inside the middle propagation path is modeled as

(4) $$A_{i+1}=g\left(A_{i},D_{i},E_{i}\right)$$

where $A_{i}$ denotes the internal adaptive timing state of the recursive middle path at stage *i*, $D_{i}$ represents the deterministic delay contribution associated with the current challenge-controlled routing decision, and $E_{i}$ represents the bounded entropy contribution applied at that stage. The function g(·) is not a software-computed function; rather, it represents the hardware-dependent state transition produced by the recursive interconnections, path-selection logic, and entropy-controlled timing elements. Consequently, the timing state of stage i+1 depends on both the current challenge decision and the propagation history accumulated through earlier stages. This history-dependent behavior increases the nonlinearity of the challenge–response mapping and makes the response more difficult to approximate using conventional linear or regression-based models. The arbiter positioned at the output stage of the proposed RHE-PUF determines the final response bit according to the relative propagation delays between competing paths as

(5) $$R=\left\{\begin{array}{cc} 1, & \mathrm{if}\;\tau_{u}>\tau_{l}\\ 0, & \mathrm{otherwise} \end{array}\right.$$

where $\tau_{u}$ and $\tau_{l}$ denote the cumulative delays of the upper and lower propagation paths, respectively. The recursive adaptive middle channel continuously perturbs both propagation paths, thereby producing highly nonlinear and unpredictable response behavior. To further enhance unpredictability and anti-modeling robustness, the proposed architecture integrates dedicated entropy injection modules $E_{1}$ and $E_{2}$, as shown in Figure 3. As a simple illustrative example, consider two challenges that differ in only one bit at an early propagation stage. In a conventional Arbiter PUF, this bit primarily changes the direct or crossed routing decision, and the resulting delay difference may remain approximately linear with respect to the challenge. In the proposed RHE-PUF, the same routing decision also changes the adaptive state of the recursive middle path. When the signal reaches an entropy-injection node, such as $E_{1}$, a bounded timing perturbation is applied to the recursive state before it influences a later propagation stage. Consequently, the delay contribution at the later stage depends on the current challenge bit, the earlier propagation history, and the entropy-controlled state. This history-dependent interaction makes the overall challenge–response mapping more nonlinear. The perturbation is limited so that it modifies the relative path timing without dominating the intrinsic device-specific delay difference, thereby preserving response repeatability. These entropy nodes introduce controlled stochastic perturbations into selected propagation stages without compromising response stability. Consequently, the generated CRPs become temporally dynamic and statistically decorrelated, thereby significantly increasing resilience against machine learning approximation attacks such as SVMs, LR, Covariance Matrix Adaptation Evolution Strategies (CMA-ES), and DNNs. After generating the hardware-rooted response, the proposed framework transforms the CRP output into a temporary anonymous identity token to support privacy-aware UPnP discovery operations. Conventional UPnP protocols expose static device identifiers during SSDP discovery broadcasts, enabling adversaries to track devices and infer user activities. Equation (5) represents the output of a single RHE-PUF evaluation and therefore produces one response bit. For authentication, a response vector of length $L_{R}$ is constructed by applying a sequence of $L_{R}$ distinct challenges,

(6) $$R=\left[R\left(C_{1}\right),R\left(C_{2}\right),\ldots ,R\left(C_{L_{R}}\right)\right],$$

where each $R\left(C_{j}\right)\in \left\{0,1\right\}$ is generated according to Equation (5). The resulting vector $R\in {\left\{0,1\right\}}^{L_{R}}$ is the response used for enrollment, helper-data generation, cryptographic hashing, and subsequent session-key derivation. The role of the entropy nodes is to modify the effective timing state of selected propagation stages rather than to generate an unconstrained random response. The injected perturbation is therefore limited relative to the intrinsic delay difference between the competing paths. This bounded perturbation increases the diversity and nonlinearity of the observed challenge–response behavior while reducing the probability that environmental or entropy variations repeatedly move the arbiter decision across its threshold. The measured reliability is used to evaluate whether this balance between response unpredictability and repeatability is maintained under the tested operating conditions. To eliminate this vulnerability, the proposed framework generates dynamic ephemeral identities according to

(7) $$T_{i}=H(R_{i}\;\|\;N_{i}\;\left\|\;t\right)$$

where H(·) denotes a cryptographic hash function, $R_{i}$ is the generated PUF response, $N_{i}$ denotes a nonce value, and *t* represents the temporal synchronization parameter. Since the generated token changes dynamically with time and nonce updates, long-term device tracking and behavioral correlation attacks become computationally impractical. As illustrated in Figure 2, the generated ephemeral identity token is forwarded to the Privacy-Aware Discovery Engine, which performs anonymous authentication and secure service orchestration. Instead of exposing raw device descriptors and service metadata, the discovery engine validates token authenticity before allowing participation in the UPnP discovery process. The authentication verification process is defined as

(8) $$\Gamma =\left\{\begin{array}{cc} 1, & \mathrm{if}\;T_{i}\in V\\ 0, & \mathrm{otherwise} \end{array}\right.$$

where V denotes the set of valid authenticated ephemeral tokens. Only authenticated anonymous entities are permitted to exchange discovery information and request service enumeration. The protected service advertisement requires a session or authorized-group key *K*. In the proposed system model, this key is established only after successful PUF-based authentication. The trusted verifier first validates the response generated for the issued challenge. After successful validation, the authorized participants obtain or derive the session key through an authenticated key-establishment procedure. The raw PUF response is not transmitted in the SSDP advertisement and is not used as a permanent public identifier. A possible deployment can derive the session key from authenticated session material as

(9) $$K=\mathrm{KDF}\left(R_{i}\;\|\;N_{i}^{\left(d\right)}\;\left\|\;N_{i}^{\left(v\right)}\;\right\|\;t\right),$$

where KDF(·) denotes a cryptographic key-derivation function, $R_{i}$ is the validated PUF response, $N_{i}^{\left(d\right)}$ and $N_{i}^{\left(v\right)}$ are fresh nonces generated by the device and verifier, respectively, and *t* is a session-validity parameter. Alternatively, a trusted authority may distribute *K* through an authenticated secure channel. The current implementation evaluates protected advertisement processing and PUF-assisted authentication but does not claim experimental validation of a complete network-wide key-management, revocation, or group-rekeying protocol. The Privacy Preservation Layer further incorporates traffic obfuscation and metadata protection mechanisms to eliminate information leakage during SSDP communication. Traditional UPnP advertisements reveal sensitive device attributes including device type, manufacturer information, firmware versions, and supported services. After session-key establishment, the sensitive portion of the service advertisement is protected as

(10) $$M_{s}=E_{K}\left(T_{i}\;\left\|\;S_{i}\;\right\|\;P_{i}\right),$$

where $E_{K}\left(\cdot \right)$ denotes symmetric encryption under the session or authorized-group key *K*, $T_{i}$ is the ephemeral identity token, $S_{i}$ represents the protected service descriptor, and $P_{i}$ contains the associated access-policy information. The protected message therefore hides the permanent device identity and sensitive service metadata from unauthorized listeners. The proposed framework does not replace the SSDP multicast transport. Instead, it introduces a secure encapsulation layer for the identity- and service-sensitive content of the advertisement. The outer message retains the information required for multicast delivery and protocol parsing, while the ephemeral identity, protected service descriptor, and access-policy attributes are carried within the encrypted payload. An authorized discovery node first verifies the freshness and validity of the associated ephemeral token. It then obtains or derives the corresponding key *K* and decrypts the protected advertisement. After successful decryption and integrity verification, the permitted service information is passed to the normal UPnP service-selection process. Unauthorized listeners may observe the multicast transmission but cannot recover the protected identity or service metadata. Thus, the proposed design extends SSDP through secure payload encapsulation rather than replacing it with an unrelated discovery protocol, as shown in Figure 2. The exact packet-field allocation and interoperability profile would need to be standardized for deployment across heterogeneous UPnP implementations. The trust score of device *i* is calculated as

(11) $$\Psi_{i}=\lambda_{1}A_{i}^{\mathrm{auth}}+\lambda_{2}B_{i}+\lambda_{3}H_{i}$$

where $A_{i}^{\mathrm{auth}}$ denotes the normalized authentication-confidence value of device *i*, $B_{i}$ represents its normalized behavioral-consistency value, and $H_{i}$ represents its normalized historical trust value. The coefficients $\lambda_{1}$, $\lambda_{2}$, and $\lambda_{3}$ determine the relative importance of these components and satisfy

(12) $$\lambda_{1}+\lambda_{2}+\lambda_{3}=1,\;\lambda_{1},\lambda_{2},\lambda_{3}\geq 0.$$

The weights are deployment-dependent policy parameters. A higher value of $\lambda_{1}$ emphasizes successful hardware authentication, while larger values of $\lambda_{2}$ or $\lambda_{3}$ place greater emphasis on current behavioral consistency or historical observations, respectively. Devices whose trust score falls below a predefined security threshold are automatically isolated from the discovery infrastructure. The proposed framework establishes a fundamentally new hardware-assisted security paradigm for UPnP ecosystems. By integrating recursive entropy-enhanced PUF authentication, anonymous ephemeral identity generation, privacy-aware service orchestration, adaptive trust verification, and encrypted resource discovery into a unified architecture, the proposed design simultaneously addresses authentication security, privacy preservation, anti-spoofing protection, anti-tracking defense, and secure resource enumeration. Consequently, the proposed architecture provides a lightweight, scalable, and highly resilient security solution suitable for next-generation IoT, edge computing, and smart home UPnP environments.

### 3.1. Enrollment and Authentication Procedure

To establish a complete hardware-rooted trust framework, the proposed RHE-PUF architecture employs two operational phases, namely the Enrollment Phase and the Authentication (Validation) Phase, as shown in Algorithm 1. Unlike conventional software-based authentication mechanisms that permanently store cryptographic secret keys inside non-volatile memory, the proposed framework never stores the secret response generated by the PUF. Instead, authentication relies on dynamically generated CRPs, thereby eliminating secret key extraction attacks.
**Algorithm 1** Enrollment and Authentication Procedure.  1: Generate random challenge $C_{i}$  2: Evaluate RHE-PUF  3: Compute response $R_{i}$  4: Repeat evaluation *m* times  5: Generate helper data $H_{i}$  6: Store $\left(C_{i},H_{i},h\left(R_{i}\right)\right)$  7: **for** each authentication request **do**  8:     Send unused challenge $C_{j}$  9:     Generate response $R_{j}$ 10:     Reconstruct ${\hat{R}}_{j}$ 11:     **if** $h\left({\hat{R}}_{j}\right)=h\left(R_{j}\right)$ **then** 12:        Generate temporary identity 13:        Allow secure UPnP discovery 14:     **else** 15:        Reject authentication 16:     **end if** 17: **end for**


#### 3.1.1. Entropy Source Generation

The entropy injection nodes $E_{1}$ and $E_{2}$ shown in Figure 3 are implemented using two independent ring-oscillator-based true random number generators (RO-TRNGs). Each entropy source consists of multiple free-running ring oscillators operating at slightly different frequencies due to intrinsic manufacturing variations. Thermal noise, oscillator phase jitter, and metastability are sampled using asynchronous counters to generate unbiased random bits. To further improve statistical randomness, the sampled bits are processed using a lightweight Von Neumann whitening circuit before being injected into the adaptive delay stages of the proposed RHE-PUF. Consequently, the injected entropy continuously perturbs the delay propagation characteristics, producing session-dependent nonlinear delay behavior that significantly increases resistance against machine-learning-based modeling attacks.

#### 3.1.2. Enrollment Phase

The enrollment phase is executed only once during device provisioning in a trusted manufacturing or deployment environment. During this phase, the authentication server communicates directly with the embedded RHE-PUF implemented on the Xilinx Spartan-7 FPGA. A set of randomly generated challenges

(13) $$C=\{c_{1},c_{2},\ldots ,c_{N}\}$$

is applied to the proposed RHE-PUF. For each challenge, the PUF generates a unique response

(14) $$R_{i}=f_{PUF}\left(c_{i}\right)$$

where $f_{PUF}\left(\cdot \right)$ denotes the nonlinear transfer function implemented by the proposed recursive entropy-enhanced delay architecture. Since environmental variations may slightly affect the raw response, each challenge is evaluated multiple times during enrollment. A majority-voting mechanism is employed to generate a stable reference response

(15) $$R_{i}^{*}=Majority\left(R_{i}^{1},R_{i}^{2},\ldots ,R_{i}^{m}\right)$$

where *m* denotes the number of repeated measurements. After repeated measurements and majority voting, the stabilized response vector $R^{*}$ is processed by the helper-data generation function,

(16) $$\left(P,K\right)=\mathrm{Gen}\left(R^{*}\right),$$

where *P* denotes the public helper data and *K* is the stable secret derived from the enrolled response. The verifier stores the challenge-set identifier, the helper data *P*, and the cryptographic verification value h(K). Neither the raw response vector $R^{*}$ nor the reconstructed secret *K* is stored in plaintext.

#### 3.1.3. Authentication (Validation) Phase

Whenever a UPnP device attempts to advertise or discover services, the authentication server first initiates a hardware authentication session. A fresh random challenge $C_{j}\in C$ is selected and transmitted to the requesting device. The embedded RHE-PUF generates a new response

(17) $$R_{j}=f_{PUF}\left(C_{j}\right).$$

During authentication, the same challenge set is applied to obtain the noisy response vector $R^{\prime }$. The reconstruction function combines $R^{\prime }$ with the stored helper data *P* to recover

(18) $$K^{\prime }=\mathrm{Rep}\left(R^{\prime },P\right).$$

The final authentication decision is based on

(19) $$h\left(K^{\prime }\right)=h\left(K\right).$$

If the equality holds, authentication succeeds; otherwise, the request is rejected. After successful authentication, the reconstructed secret $K^{\prime }$ is used to derive the temporary anonymous identity

(20) $$ID_{\mathrm{temp}}=SHA256(K^{\prime }\;\|\;T\;\|\;Nonce),$$

where *T* denotes the current timestamp and Nonce is a fresh random value generated for each authentication session, thereby preventing long-term device tracking and replay attacks. The helper-data generation algorithm follows the fuzzy extractor paradigm. During enrollment, the function

(21) (P,K)=Gen(R)

produces a public helper string *P* and a stable cryptographic key *K*. During authentication,

(22) $$K^{\prime }=Rep\left(R^{\prime },P\right)$$

reconstructs the identical key from the noisy response $R^{\prime }$. Since the helper data contains only redundancy information, it does not reveal useful information regarding the original PUF response. The reconstructed PUF key $K^{\prime }$ is not directly used as a communication encryption key; instead, a SHA-256-based key derivation procedure is employed to generate fresh session-specific cryptographic material. In particular, an encryption key $K_{\mathrm{enc}}$ is derived from $K^{\prime }$, the session nonce, timestamp, and session identifier, while separate cryptographic contexts are used to prevent key reuse across different protocol functions. The protected UPnP/SSDP payloads are encrypted using AES-256 in Galois/Counter Mode (AES-256-GCM), which simultaneously provides confidentiality and authenticated integrity through the generated authentication tag. A fresh 96-bit nonce is generated for every encryption operation and is never reused with the same session key. The nonce, timestamp, session identifier, and message type are included as authenticated protocol metadata, while the encrypted UPnP payload is carried as the ciphertext. Replay protection is provided by validating the timestamp against a predefined acceptance window of ΔT=30 s, maintaining the session nonce history, and rejecting previously observed nonces, expired messages, invalid authentication tags, or messages associated with terminated sessions. Following successful PUF validation, the derived session key and temporary identity remain valid only for the current authenticated session and are securely discarded when the session expires or terminates; a new PUF authentication, fresh nonce, and fresh key derivation are therefore required for subsequent sessions. Consequently, no permanent copy of the reconstructed PUF key, encryption key, or session key is stored, while the verifier retains only the enrolled challenge/reference information and public helper data required for PUF reconstruction and validation.

#### 3.1.4. Challenge Management

To prevent challenge replay and machine learning attacks, each challenge is used only once. The authentication server maintains a challenge pool and permanently removes each challenge after successful authentication. Furthermore, challenge selection follows a cryptographically secure pseudo-random scheduling mechanism, ensuring that future challenges cannot be predicted by adversaries. Even if an attacker captures previous communication sessions, previously observed CRPs cannot be reused because every authentication employs a fresh challenge together with a newly generated timestamp and nonce.

#### 3.1.5. Protection of CRPs and PUF Security

The proposed authentication framework is designed to protect the confidentiality of CRPs while establishing a hardware-rooted trust model suitable for lightweight UPnP-enabled IoT devices. Unlike conventional strong PUF-based authentication systems that maintain large databases of raw CRPs, the proposed framework never stores or transmits the original PUF responses. During the enrollment phase, only the challenge index, public helper data, and the cryptographic hash of the stabilized reference response are stored in the authentication server. The helper data is generated using a fuzzy extractor and contains only redundancy information required for response reconstruction during authentication. Since the helper data is mathematically independent of the original response, it does not reveal useful information about the underlying PUF characteristics and therefore cannot be exploited to reconstruct the secret hardware fingerprint. Consequently, permanent exposure of sensitive CRPs is eliminated, significantly reducing the attack surface associated with database leakage and memory extraction attacks.

To further enhance authentication reliability under process, voltage, and temperature (PVT) variations, the proposed RHE-PUF employs repeated response sampling followed by majority voting during the enrollment stage to generate a stable reference response. During authentication, the received response is reconstructed using the stored helper data and verified through a fuzzy extractor reconstruction function. Instead of requiring an exact bit-by-bit match, authentication is performed using a configurable Hamming-distance threshold,

(23) $$HD(\hat{R},R^{*})\leq \tau ,$$

where HD(·) denotes the Hamming distance between the reconstructed response $\hat{R}$ and the enrolled reference response $R^{*}$, while τ represents the maximum allowable bit mismatch determined during system calibration. This threshold-based verification improves robustness against environmental noise. The authentication threshold is selected experimentally to achieve an appropriate balance between reliability and security.

The proposed architecture further protects the authentication protocol against replay, impersonation, and long-term tracking attacks through dynamic challenge management and ephemeral identity generation. Every authentication session employs a fresh challenge selected from a cryptographically secure challenge pool, and each challenge is permanently retired after successful verification. As a result, previously observed CRPs cannot be reused by an adversary to impersonate a legitimate device. After successful authentication, both the device and the authentication server independently derive an identical session key from the reconstructed PUF response, timestamp, and nonce. This session key is subsequently used to generate a temporary authentication token and anonymous device identity that remain valid only for the current UPnP discovery session. Since both communicating entities independently compute the same token without transmitting the PUF response over the network, token verification can be performed securely without maintaining a database of raw CRPs or exposing hardware secrets to the communication channel.

## 4. Experimental Setup and Results

This section presents a comprehensive hardware implementation and experimental validation of the proposed RHE-PUF-based secure UPnP framework. The complete architecture was fully implemented on a Xilinx Spartan-7 XC7S50 FPGA platform to evaluate its practicality, scalability, security robustness, and suitability for resource-constrained IoT and edge computing environments. Unlike software-based UPnP protection mechanisms that rely on operating system security primitives and software-managed credentials, the proposed framework introduces a fully hardware-oriented trust establishment and privacy preservation architecture capable of providing intrinsic device authentication and anonymous resource discovery directly at the hardware level.

The proposed RHE-PUF is implemented as a challenge-controlled, multi-path delay architecture consisting of three parallel propagation paths, namely an upper delay path, an adaptive middle path, and a lower delay path. The implemented RHE-PUF uses a 4-bit challenge ($N_{c}=4$) and four delay stages per propagation path ($N_{s}=4$), with each challenge bit controlling the corresponding delay-selection operation. The three propagation paths are excited simultaneously by the applied challenge, and selected intermediate stages are cross-coupled to introduce additional dependencies among the paths. In particular, intermediate signals from the upper path are coupled to subsequent stages of the adaptive middle path, while selected middle-path states are recursively fed toward later stages of the upper and lower paths. Two entropy injection sources, $E_{1}$ and $E_{2}$, are inserted at different locations of the upper delay network to dynamically perturb the propagation behavior. These entropy sources, together with the recursive feed-forward and adaptive cross-coupling structures, make the final response dependent on the joint behavior of the three propagation paths rather than on a simple additive delay relationship. At the end of the delay network, the resulting path states are supplied to the arbiter, which determines the final response according to the relative arrival behavior of the competing signals. The generated response is subsequently sampled and supplied internally to the authentication subsystem for response stabilization, verification, session-key derivation, and ephemeral-token generation. Importantly, the raw PUF response is never transmitted through the UPnP/SSDP communication interface.

Because physical routing asymmetry can substantially influence Arbiter-PUF behavior, particular attention was given to FPGA placement and routing during implementation. The RHE-PUF was implemented with the four corresponding delay stages of the upper, middle, and lower paths physically constrained to aligned FPGA regions to minimize unintended systematic routing imbalance. Equivalent stages across the three competing paths were assigned to neighboring or equivalent programmable resources, while the recursive interconnections and two entropy-injection circuits were preserved during synthesis and implementation to prevent logic optimization from modifying the intended PUF structure. The routing constraints were selected to maintain comparable inter-stage interconnect structures while retaining the intrinsic manufacturing-dependent delay variations required for PUF uniqueness. The same RTL description, synthesis settings, timing constraints, placement strategy, and routing constraints were applied throughout the experimental evaluation. Consequently, the measured CRPs were obtained from the actual placed-and-routed FPGA implementation rather than from an idealized RTL simulation.

### 4.1. Experimental Setup

The proposed framework was designed using Verilog HDL and synthesized using the Xilinx the Spartan-7 XC7S50 FPGA device. The entire security architecture, including the Recursive Hybrid Entropy PUF engine, challenge–response authentication subsystem, ephemeral identity generator, encrypted SSDP communication controller, adaptive trust verification module, and privacy-aware UPnP discovery engine, was implemented entirely within the FPGA fabric. The FPGA operated at a system clock frequency of 100 MHz while emulating multiple heterogeneous UPnP-enabled smart devices and services. Specifically, the implemented UPnP traffic emulator synthesized realistic discovery behaviors corresponding to Belkin WeMo smart switches and Texas Instruments temperature sensing services. The WeMo emulation layer generated dynamic ON/OFF control advertisements, device status notifications, and secure resource descriptors, while the temperature sensing emulator generated periodic environmental telemetry advertisements integrated into the proposed secure discovery framework.

The proposed architecture continuously generated encrypted SSDP advertisements and anonymous discovery tokens while simultaneously processing authentication requests and trust verification operations in real time. To evaluate environmental stability and robustness, the FPGA platform was experimentally tested under varying operating conditions including dynamic voltage scaling and thermal variations ranging from 20 °C to 75 °C. Furthermore, the experimental environment continuously injected malicious SSDP packets, replay traffic, service enumeration requests, and spoofing advertisements to evaluate real-time security behavior. The proposed attack emulation engine also generated large CRP datasets used for machine learning modeling attacks. More than $10^{6}$ CRPs were collected during the evaluation process to investigate the resistance of the proposed architecture against regression-based and deep learning-based approximation attacks.

The proposed framework is viewed as a secure extension to the conventional UPnP discovery protocol rather than a replacement for the UPnP standard. The standard SSDP multicast addresses and the UPnP discovery workflow are preserved to maintain interoperability with existing UPnP service discovery procedures. However, the payload of the SSDP advertisement is extended by replacing sensitive device identifiers and service descriptors with authenticated ephemeral identifiers generated from the RHE-PUF authentication framework. Consequently, devices participating in the secure discovery framework must implement the proposed authentication extension to interpret the protected advertisements and perform mutual authentication. To support heterogeneous IoT deployments containing legacy UPnP devices, the proposed architecture may optionally employ a secure discovery gateway that translates between standard UPnP discovery messages and the proposed secure advertisements. In this deployment mode, legacy devices continue to communicate using conventional SSDP messages, whereas secure devices exchange authenticated advertisements through the proposed protocol without exposing permanent device identities. Therefore, the proposed framework remains compatible with the standard UPnP discovery sequence while extending the discovery payload with additional authentication information. Since the multicast discovery procedure, SSDP message exchange sequence, and service discovery state machine remain unchanged, the proposed approach can be incrementally deployed without requiring modifications to the underlying UPnP communication architecture.

To ensure the reproducibility of the experimental evaluation, the proposed framework was implemented and validated on three FPGA development boards, each programmed with the same hardware configuration and synthesized using identical implementation constraints. For each FPGA board, approximately $3.5\times 10^{5}$ valid CRPs were collected, resulting in a total dataset exceeding $10^{6}$ CRPs for security evaluation and machine-learning attack analysis. All experiments were conducted under a regulated core supply voltage ranging from 0.95V to 1.05V with temperature varied from 20 °C to 75 °C in 5 °C intervals using a programmable environmental chamber. At each voltage-temperature operating point, the authentication and discovery experiments were repeated 100 times to evaluate response reliability, authentication consistency, and environmental robustness. The UPnP traffic generator emulated a mixed smart-home environment consisting of Belkin WeMo switch advertisements and Texas Instruments temperature sensor services, producing SSDP M-SEARCH requests and NOTIFY advertisements with configurable inter-arrival times ranging from 50 to 500 ms. During each experimental run, both legitimate and malicious traffic streams were generated concurrently, including replay attempts, spoofed advertisements, unauthorized service discovery requests, and high-rate service enumeration attacks, enabling comprehensive evaluation of the proposed framework under realistic network conditions.

### 4.2. Hardware Resource Utilization

The FPGA implementation results are summarized in Table 1, where the proposed framework is compared with two hardware baselines: (i) a conventional Arbiter PUF implementation and (ii) a standard UPnP discovery engine without hardware security. This comparison enables quantification of the hardware overhead introduced by the recursive entropy generation mechanism, privacy-preserving authentication modules, and secure UPnP discovery engine. As expected, the proposed architecture requires additional logic resources due to the integration of adaptive delay stages, entropy injection circuits, fuzzy-extractor-based authentication, ephemeral identity generation, and encrypted SSDP processing. Nevertheless, the overall resource utilization remains modest relative to the available resources of the FPGA, occupying only a small fraction of the programmable logic while maintaining a maximum operating frequency of 192 MHz.

Compared with the baseline Arbiter PUF, the increase in LUTs and flip-flops is primarily attributed to the recursive feed-forward network, adaptive cross-coupling structure, helper-data processing, and session-token generation modules. Similarly, relative to the conventional UPnP discovery engine, the additional hardware originates from the integrated authentication engine, privacy-preserving service advertisement logic, and encrypted discovery controller. Despite these enhancements, the measured dynamic power remains below 1 W and the authentication latency is only 0.88 μs, demonstrating that the additional hardware overhead is modest considering the substantial improvements in security, anonymity, and resistance to replay, impersonation, and machine-learning attacks. These results confirm that the proposed architecture provides an effective trade-off between hardware cost and security, making it suitable for deployment in resource-constrained FPGA-based IoT edge devices.

### 4.3. Statistical Evaluation of the Proposed RHE-PUF

**Figure 4:**
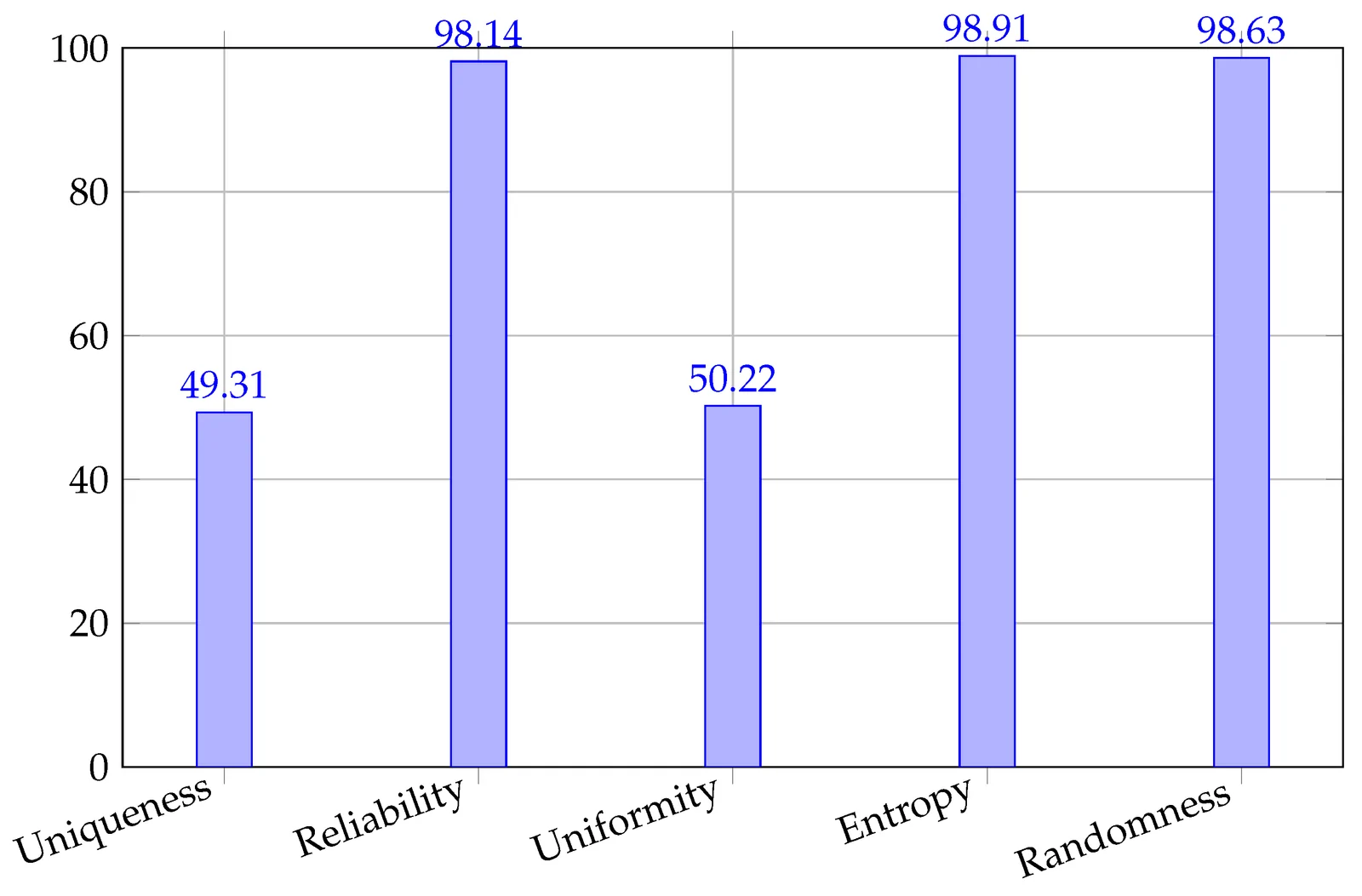
Statistical characteristics of the proposed RHE-PUF architecture.

The quality of the generated challenge–response pairs was evaluated using standard PUF statistical metrics including uniqueness, reliability, uniformity, entropy, and randomness. Figure 4 presents the measured statistical characteristics of the proposed RHE-PUF architecture. The proposed architecture achieved an inter-device uniqueness of 49.31%, which is extremely close to the ideal value of 50%. This indicates strong device distinguishability and highly independent CRP generation behavior across different hardware instances. Furthermore, the measured uniformity of 50.22% demonstrates balanced response distributions between logical zeros and ones, thereby minimizing statistical bias. The reliability exceeded 98% under varying environmental conditions, confirming stable response regeneration despite thermal fluctuations and supply voltage variations. The enhanced entropy and randomness properties originated from the recursive adaptive delay coupling and entropy injection stages integrated into the proposed architecture. Unlike conventional arbiter PUFs that exhibit partially linear propagation characteristics, the proposed recursive entropy-driven structure continuously perturbs internal timing dynamics, thereby substantially increasing CRP unpredictability. The reported values represent the aggregate measurements obtained under the evaluated experimental conditions. Device-level repeated measurements required to calculate statistically reliable standard deviations, variances, and confidence intervals were not available in the current experimental records. Therefore, these dispersion measures are not reported to avoid presenting unsupported statistical estimates. Similarly, the reported reliability of 98.14% summarizes response consistency across the evaluated voltage and temperature variations. A detailed reliability matrix reporting the response error at each individual voltage and temperature operating point is left for future experimental evaluation.

### 4.4. Secure UPnP Discovery Performance Analysis

**Figure 5:**
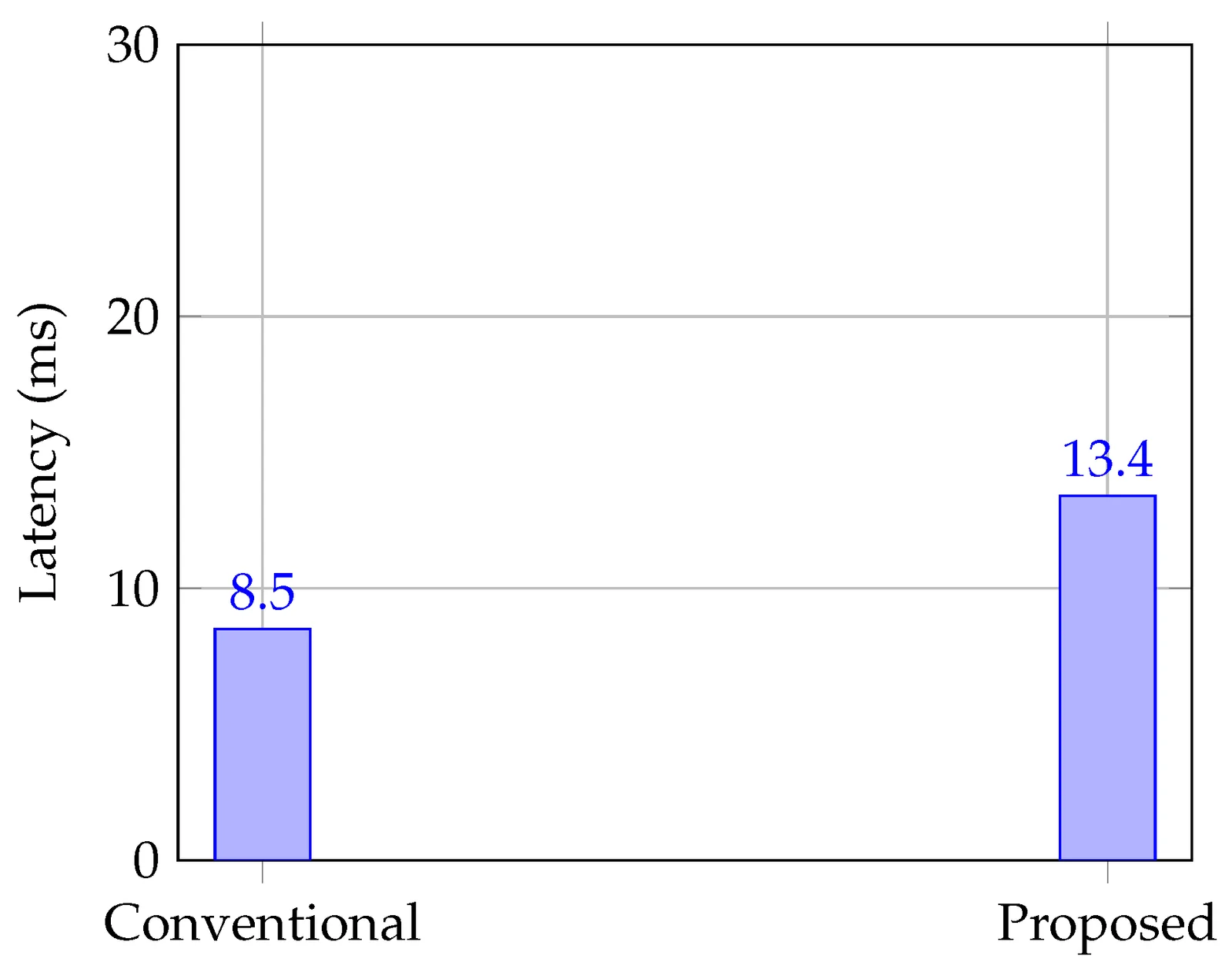
Comparison of UPnP discovery latency.

The proposed framework was evaluated using synthesized UPnP discovery traffic corresponding to smart switch services and temperature sensing advertisements. The experiments measured authentication delay, encrypted SSDP processing overhead, service enumeration latency, and anonymous discovery response time. Figure 5 compares the discovery latency of conventional UPnP discovery and the proposed secure discovery architecture. Although the proposed architecture introduced additional cryptographic operations and authentication stages, the overall discovery latency remained sufficiently low for real-time IoT applications. The measured latency overhead primarily originated from ephemeral identity generation, encrypted SSDP packet formation, and trust verification operations. The achieved processing delay remained significantly lower than the latency tolerance requirements of typical smart home and edge computing environments, confirming the practical feasibility of the proposed framework. The proposed security mechanisms increase the measured discovery delay from 8.5 ms for conventional UPnP to 13.4 ms, corresponding to an additional overhead of 4.9 ms. This overhead results from PUF-assisted authentication, ephemeral identity generation, encrypted SSDP advertisement processing, and trust verification. UPnP discovery is a control-plane operation performed when devices advertise or locate services and is not part of the continuous time-critical sensing or actuation data path. Therefore, the measured delay remains acceptable for the evaluated smart-home and edge-IoT discovery scenarios. Nevertheless, application-specific latency requirements should be considered when deploying the framework in systems with strict real-time constraints.

### 4.5. Machine Learning Modeling Attack Evaluation

**Figure 6:**
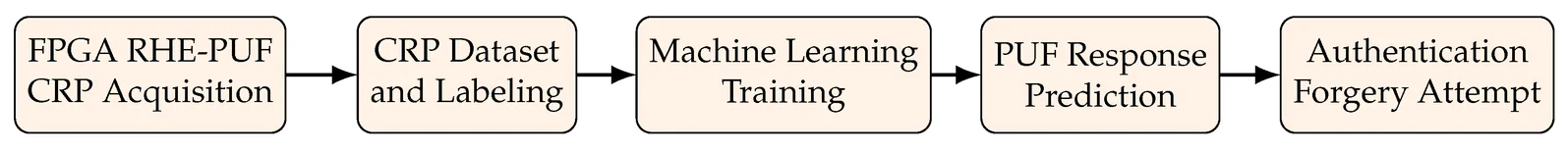
Machine-learning modeling attack workflow using CRPs acquired directly from the implemented RHE-PUF.

To investigate resistance against hardware modeling attacks, several machine learning algorithms, including LR, SVM, RF, and DNNs, were trained using large CRP datasets generated directly from the FPGA implementation. The machine-learning attack workflow is illustrated in Figure 6. The CRP dataset was acquired directly from controlled challenge–response evaluations of the implemented RHE-PUF on the Spartan-7 FPGA. This CRP acquisition process was performed independently of the encrypted UPnP/SSDP traffic evaluation. The collected CRPs were then used to train the machine-learning models and evaluate their ability to predict previously unseen PUF responses. The experimental results demonstrate that the proposed recursive entropy-enhanced architecture significantly outperformed conventional arbiter PUFs in terms of anti-modeling robustness. Even the DNN-based attack achieved only 58.27% prediction accuracy. The substantial security improvement originated from the recursive adaptive propagation structure and entropy injection mechanisms, which continuously altered internal propagation dynamics and eliminated linear CRP relationships commonly exploited by machine learning algorithms.

To ensure reproducibility, all machine learning attacks were performed using the same CRP dataset collected from the implemented Spartan-7 FPGA. A total of $1.0\times 10^{6}$ valid challenge–response pairs were acquired, of which 80% were randomly selected for training and the remaining 20% were reserved for testing. The reported prediction accuracies correspond to the average of ten independent experimental runs using different random seeds to eliminate initialization bias. Hyperparameters for each learning algorithm were selected through five-fold cross-validation using the training dataset. LR employed an $L_{2}$ regularization parameter of C=1.0, while the SVM used a radial basis function (RBF) kernel with C=10 and γ=0.01. The RF classifier consisted of 200 decision trees with a maximum tree depth of 20. The DNN contained four fully connected hidden layers comprising 512, 256, 128, and 64 neurons, respectively, using ReLU activation, the Adam optimizer with a learning rate of $10^{-3}$, a batch size of 256, and 100 training epochs.

### 4.6. Replay and Spoofing Attack Evaluation

**Figure 7:**
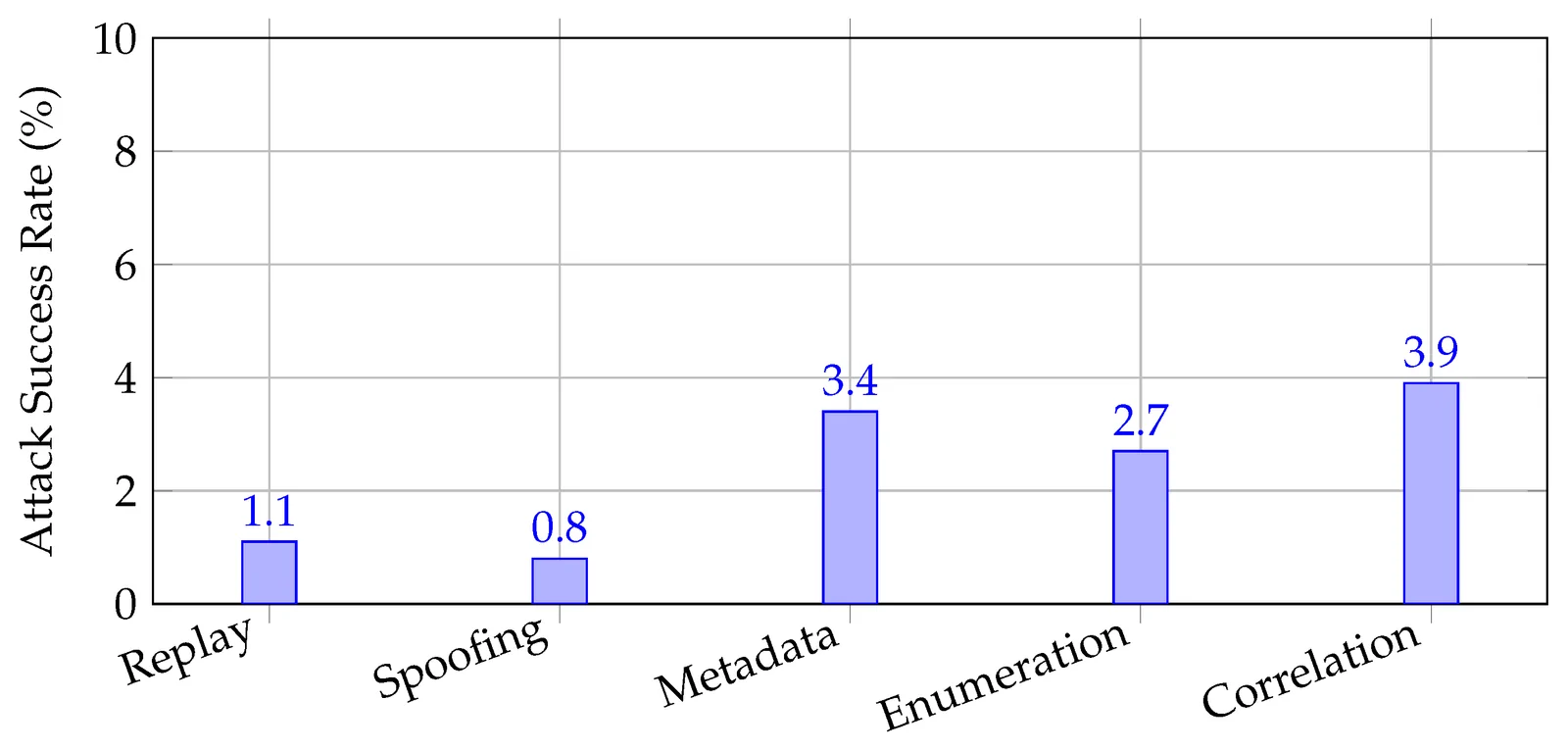
Attack success rates against the proposed framework.

For each attack category, the attack success rate is defined as

(24) $$\mathrm{ASR}=\frac{N_{\mathrm{accepted}}^{\mathrm{malicious}}}{N_{\mathrm{attempted}}^{\mathrm{malicious}}}\times 100\%,$$

where $N_{\mathrm{attempted}}^{\mathrm{malicious}}$ is the total number of injected malicious attempts and $N_{\mathrm{accepted}}^{\mathrm{malicious}}$ is the number of those attempts that were incorrectly accepted as valid by the proposed framework. A total of 1000 independent attack attempts were performed for each attack category shown in Figure 7. A replay attack is considered successful when a previously captured SSDP advertisement or authentication token is accepted after retransmission. A spoofing attack is considered successful when a forged advertisement or impersonated device identity passes the authentication and discovery validation procedure. A metadata attack is considered successful when an adversary extracts sufficient protected metadata from the observed discovery traffic to correctly identify the corresponding device or service. A service-enumeration attack is considered successful when an unauthorized request obtains a valid service descriptor or resource identifier. Finally, a traffic-correlation attack is considered successful when the adversary correctly associates two or more temporally separated discovery messages with the same physical device using the observable network characteristics.

Replay and spoofing attacks were experimentally emulated using the integrated FPGA attack engine. During the replay attack scenario, previously captured encrypted SSDP advertisements were retransmitted into the discovery network after token expiration and with previously observed nonces. Spoofing attempts used forged device identities, invalid authentication tokens, and modified service-advertisement fields. Metadata, enumeration, and correlation attacks were similarly generated by injecting malformed or unauthorized discovery requests and by analyzing observable packet-level characteristics, respectively. Each attack attempt was independently classified as either accepted or rejected according to the authentication, token-validation, access-policy, and discovery-validation outcomes of the proposed architecture. The corresponding numbers of successful malicious attempts in the 1000-trial experiments were 11, 8, 34, 27, and 39 for replay, spoofing, metadata, enumeration, and correlation attacks, respectively, resulting in attack success rates of 1.1%, 0.8%, 3.4%, 2.7%, and 3.9%. A replayed packet was therefore counted as successful only when it was accepted after its original authentication session had expired, whereas spoofing, metadata, enumeration, and correlation attacks were counted as successful only when the attacker achieved the corresponding unauthorized objective defined above. Since the proposed framework continuously generates dynamic ephemeral identities, previously captured authentication material cannot be directly reused in a subsequent session. The replay resistance property can be mathematically represented as

(25) $$T_{i}\left(t_{1}\right)\neq T_{i}\left(t_{2}\right),\;t_{1}\neq t_{2}$$

where $T_{i}\left(t\right)$ denotes the generated ephemeral identity at time *t*. Figure 7 presents the measured attack success rates. The consistently low values demonstrate that the combined PUF authentication, nonce-based session management, encrypted SSDP communication, and privacy-aware discovery mechanisms substantially reduce the probability of successful unauthorized operations.

### 4.7. Privacy Preservation Analysis

**Figure 8:**
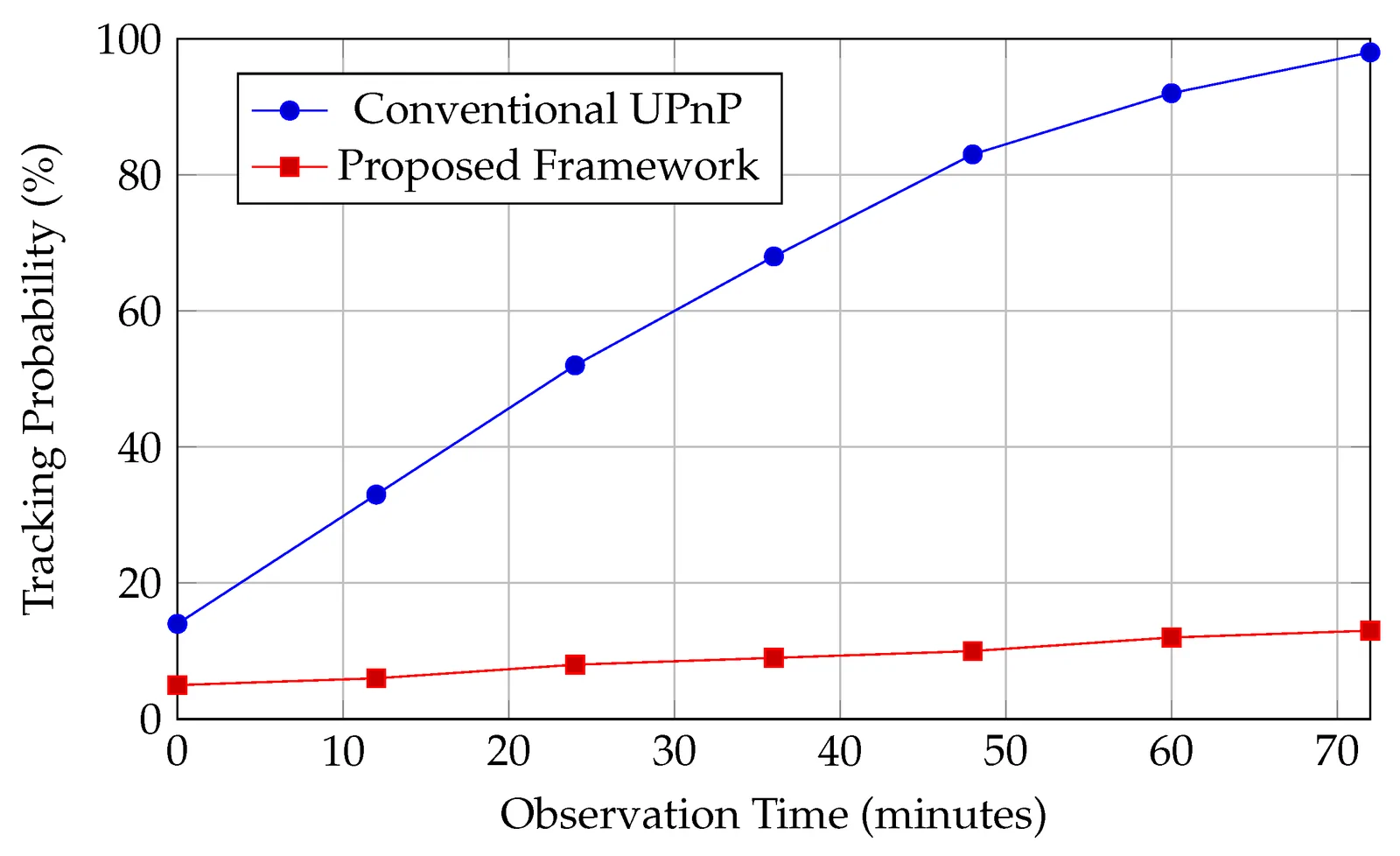
Comparison of long-term device tracking probability.

For the privacy evaluation, the attacker is modeled as a passive network observer with access to multicast SSDP traffic. The attacker can observe packet arrival times, advertisement frequency, packet size, visible protocol headers, source and destination addressing information available at the network layer, and repeated patterns in encrypted advertisements. The attacker can capture the encrypted SSDP payload but is assumed not to possess the corresponding session key and therefore cannot recover the protected ephemeral identity, service descriptor, or access-policy information. The privacy experiment used 20 emulated UPnP devices, including the WeMo switch and temperature-sensing service models, with 100 independent observation trials performed for each device at each observation interval. Thus, each point in Figure 8 represents the outcome of 2000 device-level observation instances. The observation intervals were 0, 12, 24, 36, 48, 60, and 72 min, with the same traffic-generation configuration and device population maintained throughout the experiment.

The tracking objective is to determine whether multiple observed advertisements can be associated with the same physical device over time. A tracking attempt is considered successful when the attacker establishes a stable association between a sequence of observed discovery messages and the originating device. For each observation trial, the attacker constructed a device fingerprint from the observable packet attributes, including packet timing, packet-size patterns, advertisement frequency, network-layer addressing information, and repeated encrypted-message characteristics. A tracking decision was counted as successful only when the same device could be correctly associated with its observed discovery messages across the corresponding observation interval. The tracking probability was calculated as the ratio of successful device associations to the total number of observation trials. Consequently, the reported probability represents the fraction of observation instances in which the passive observer established a stable device-to-message mapping under the evaluated traffic conditions.

The privacy-aware capability of the proposed framework was further evaluated by measuring the probability of long-term device tracking during continuous UPnP discovery operations. Conventional UPnP systems continuously expose static identifiers, enabling adversaries to correlate repeated discovery advertisements over time. In contrast, the proposed framework continuously generates dynamically changing anonymous identities, thereby significantly reducing traffic correlation capability. Figure 8 compares the device tracking probability between conventional UPnP and the proposed framework over a 72 min observation period. For each time point, the tracking probability was obtained from the 2000 observation instances generated from the 20 emulated devices and 100 independent trials per device. The conventional UPnP configuration exhibited an increasing tracking probability from 14% at the initial observation to 98% after 72 min, whereas the proposed framework increased only from 5% to 13% over the same period. These results indicate that the ephemeral identity mechanism substantially reduces the ability of a passive observer to establish stable long-term mappings between encrypted discovery-message sequences and their originating physical devices.

### 4.8. Comparison with Existing Methods

To further evaluate the effectiveness of the proposed RHE-PUF-based secure UPnP framework, a qualitative comparison was conducted with representative published PUF-based IoT authentication and FPGA security architectures. The comparison considers hardware-rooted trust, PUF-based authentication, replay protection, resistance to ML modeling attacks, privacy preservation, FPGA validation, anonymous discovery, and lightweight implementation.

Table 2 highlights an important distinction between conventional PUF-based authentication and the proposed secure UPnP architecture. He et al. [31] targeted reliability and uniqueness improvement of Arbiter-PUFs and does not address privacy-aware service discovery or anonymous UPnP communication. Similarly, Ge et al. [32] provided hardware-rooted authentication and explicitly evaluates ML-based modeling; its primary objective is PUF security rather than privacy-preserving resource discovery or protection of UPnP/SSDP metadata. Anandakumar et al. [33] improved the characteristics of conventional PUF constructions but did not integrate anonymous service discovery, encrypted SSDP communication, or dynamic device identities. Agarwal and Joshi [34] addressed PUF response reliability under environmental variations rather than end-to-end privacy-preserving communication and discovery. Babaei et al. [35] used PUF-based authentication and explicitly investigated resilience against machine-learning attacks, making it particularly relevant to the proposed work. However, the primary objective of RESA is lightweight PUF-based authentication and reconfiguration, whereas the proposed framework extends the hardware root of trust to the UPnP discovery layer through encrypted SSDP communication, ephemeral identities, metadata protection, and privacy-aware resource matching. Yoon et al. [36] conducted their experimental evaluation using a Raspberry Pi–based testbed and wireless channel information; however, their approach does not target UPnP service discovery or anonymous SSDP advertisements. In contrast to these existing approaches, the proposed RHE-PUF framework integrates hardware-rooted authentication, recursive entropy-enhanced PUF construction, machine-learning attack resistance, dynamic ephemeral identity generation, encrypted SSDP communication, privacy-aware resource matching, and replay-resistant UPnP discovery within a unified architecture. The proposed design is implemented and evaluated on a Xilinx Spartan-7 XC7S50 FPGA. In addition to PUF-level security evaluation, the proposed framework evaluates the security of the complete discovery process, including replay, spoofing, metadata exposure, service enumeration, traffic correlation, and long-term device tracking. This system-level integration represents the main distinction from prior PUF studies, which generally concentrate on hardware authentication or PUF reliability rather than combining hardware-rooted identity with privacy-preserving UPnP resource discovery.

Table 3 compares the proposed RHE-PUF framework with representative published PUF-based security approaches while explicitly identifying the implementation platform and reported measurement conditions. The FPGA-based self-correcting PUF reported by Agarwal et al. achieved 97% reliability using an error-correction mechanism, whereas the 64-stage challenge-preprocessing Arbiter PUF reported prediction accuracy below 61.33% under its machine-learning evaluation. The hybrid PUF of Kumar et al. was experimentally implemented on a Xilinx Spartan-6 FPGA, while the proposed RHE-PUF is implemented on a Xilinx Spartan-7 XC7S50 FPGA and integrates recursive delay propagation, entropy injection, hardware-rooted authentication, and privacy-aware UPnP discovery. The proposed framework achieves 98.14% measured reliability and 58.27% DNN prediction accuracy using $10^{6}$ CRPs, while additionally providing privacy protection at the UPnP discovery layer, which is not the primary objective of the individual PUF designs included for comparison.

The experimental results conclusively demonstrate that the proposed FPGA-based RHE-PUF secure UPnP framework successfully achieves hardware-efficient implementation, strong authentication capability, enhanced privacy preservation, and high resistance against machine learning and network-oriented attacks. The recursive entropy-driven propagation architecture significantly increased CRP nonlinearity and unpredictability compared to conventional arbiter PUF implementations. Furthermore, the integration of encrypted SSDP advertisements, ephemeral anonymous identities, adaptive trust verification, and privacy-aware service discovery effectively mitigated several major UPnP vulnerabilities including replay attacks, spoofing attacks, service enumeration, metadata leakage, and long-term traffic correlation. The low hardware overhead, high operating frequency, strong anti-modeling robustness, and excellent privacy preservation characteristics demonstrate that the proposed framework provides a highly practical and scalable security solution for next-generation IoT, edge computing, and smart home UPnP ecosystems.

## 5. Conclusions

This paper presented a novel FPGA-based privacy-preserving secure UPnP framework driven by a RHE-PUF. The proposed architecture was designed to address several critical security and privacy limitations of conventional UPnP discovery systems, including spoofing attacks, replay attacks, metadata leakage, service enumeration, device fingerprinting, and machine learning-based hardware modeling attacks. Unlike traditional software-oriented UPnP protection approaches, the proposed framework establishes a lightweight hardware-rooted trust architecture capable of simultaneously providing intrinsic authentication, anonymous service discovery, and confidentiality-protected communication directly at the hardware level. The proposed RHE-PUF introduced a fundamentally new recursive entropy-enhanced delay propagation structure combining adaptive timing diversification, recursive feed-forward coupling, and entropy injection mechanisms. These features substantially increased CRP nonlinearity and unpredictability, thereby significantly improving anti-modeling robustness compared to conventional Arbiter PUF architectures. Furthermore, the integration of ephemeral identity generation and encrypted SSDP advertisements enabled secure anonymous discovery operations while preventing long-term traffic correlation and device tracking. The complete framework was fully implemented on a Xilinx Spartan-7 FPGA platform and experimentally evaluated under realistic UPnP discovery environments. Experimental results demonstrated that the proposed architecture achieved near-ideal uniqueness of 49.31%, response reliability of 98.14%, and entropy exceeding 98.91%. The implementation achieved a maximum operating frequency of 192 MHz while maintaining low dynamic power consumption of only 0.84 W, confirming suitability for resource-constrained IoT edge environments. Moreover, the proposed privacy-aware discovery mechanism successfully reduced replay and spoofing attack success rates to at or below 1.1%, while long-term device tracking probability remained below 13% during extended monitoring periods. These results confirm that the proposed framework effectively mitigates major UPnP security threats while preserving lightweight operation and low hardware overhead.

## Figures and Tables

**Table 1:** Hardware overhead comparison on Spartan-7 XC7S50 FPGA.

Hardware Metric	Baseline Arbiter PUF	Standard UPnP	Proposed Framework
LUTs	894	612	2217
FFs	641	487	1784
BRAMs	1	2	7
DSP Slices	0	2	15
Maximum Frequency (MHz)	238	205	192
Dynamic Power (W)	0.29	0.37	0.84
Static Power (W)	0.18	0.18	0.20
Authentication Latency (μs)	0.41	–	0.88
Discovery Delay (ms)	–	8.9	13.4

**Table 2:** Qualitative comparison of the proposed RHE-PUF framework with PUF-based IoT security approaches.

Method	HW Rooted	PUF-Based	Anonymous Discovery	Replay Protection	ML Attack Evaluation	Privacy Protection	FPGA Validated	Lightweight
He et al. [31]	✓	✓	×	×	Partial	×	✓	✓
Ge et al. [32]	✓	✓	×	×	✓	×	✓	✓
Anandakumar et al. [33]	✓	✓	×	×	Partial	×	✓	✓
Agarwal and Joshi [34]	✓	✓	×	×	×	×	✓	✓
Babaei et al. [35]	✓	✓	×	✓	✓	×	✓	✓
Yoon et al. [36]	✓	✓	×	✓	✓	Partial	×	✓
Proposed RHE-PUF Framework	✓	✓	✓	✓	✓	✓	✓	✓

**Table 3:** Performance comparison between the proposed RHE-PUF framework and representative existing approaches.

Method	Implementation Platform	Reliability (%)	ML Prediction Accuracy (%)	Authentication/Privacy	Reported Measurement Conditions
He et al. [31]	Xilinx FPGA	–	–	PUF-based authentication	FPGA implementation; reliability and uniqueness evaluated experimentally
Ge et al. [32]	FPGA, 64-stage APUF	–	<61.33	ML-resistant PUF	Experimental CRP acquisition and ML modeling attack
Anandakumar et al. [33]	Xilinx Spartan-6 FPGA	–	–	Hardware authentication	Hybrid RS-latch/Arbiter PUF with programmable delay lines and temporal majority voting
Agarwal et al. [34]	FPGA	97	–	IoT device authentication	Golay-code-based self-error correction; reliability evaluated under environmental variations
Babaei et al. [35]	FPGA-based IoT platform	–	–	Lightweight authentication	PUF-based reconfigurable security architecture for long-lifetime IoT devices
Yoon et al. [36]	Raspberry Pi + USRP + SRAM-PUF	–	–	Replay-resistant authentication	Physical-layer information combined with device PUF; experimental IoT testbed
Proposed RHE-PUF	Xilinx Spartan-7 XC7S50	98.14	58.27	Authentication, identity, metadata, and tracking protection	Placed-and-routed FPGA implementation; $10^{6}$ CRPs for ML evaluation

## Data Availability

Data are contained within the article.
